# The leucine-rich repeat (LRR) domain of NLRP3 is required for NLRP3 inflammasome activation in macrophages

**DOI:** 10.1016/j.jbc.2022.102717

**Published:** 2022-11-17

**Authors:** Yanhui Duan, Jihong Wang, Juan Cai, Nathan Kelley, Yuan He

**Affiliations:** Department of Biochemistry, Microbiology, and Immunology, Wayne State University, Detroit, Michigan, USA

**Keywords:** innate immunity, inflammation, inflammasome, Nod-like receptor, NLRP3, ASC, apoptosis-associated speck-like protein containing a caspase-activation and recruitment domain, GSDMD, gasdermin D, iBMDMs, immortalized mouse bone marrow-derived macrophages, LPS, lipopolysaccharide, LRR, leucine-rich repeats, m.o.i., multiplicity of infection, NACHT, NAIP, CIITA, HET-E, and TP1domain, NLRP3, NOD-like receptor family pyrin domain-containing 3, PYD, pyrin domain, SFP, S-tag, FLAG, and streptavidin-binding tag

## Abstract

The NLRP3 inflammasome is a critical component of innate immunity that defends the host from microbial infections. However, its aberrant activation contributes to the pathogenesis of several inflammatory diseases. Activation of the NLRP3 inflammasome induces the secretion of proinflammatory cytokines IL-1β and IL-18 and pyroptotic cell death. NLRP3 contains a leucine-rich repeat (LRR) domain at its C terminus. Although posttranslational modifications in this LRR domain have been shown to regulate NLRP3 inflammasome activation, the role of the entire LRR domain in NLRP3 inflammasome activation remains controversial. Here, we generated mouse macrophages that express an endogenous NLRP3 mutant lacking the LRR domain. Deletion of the LRR domain diminished NLRP3 inflammasome activation in macrophages. Furthermore, using NLRP3-deficient macrophages that are reconstituted with NLRP3 mutants lacking the LRR domain, we found that deletion of the LRR domain inhibited NLRP3 inflammasome activation. Mechanistically, deletion of the LRR domain inhibited NLRP3 self-association, oligomerization, and interaction with the essential regulator NEK7. Our results demonstrate a critical role for the LRR domain in NLRP3 inflammasome activation.

Inflammasomes are cytoplasmic multiprotein complexes that form in response to microbial infection or cell damage (1). In most cases, an inflammasome contains a pattern recognition receptor as the sensor, the adaptor protein apoptosis-associated speck-like protein containing a caspase-activation and recruitment domain (ASC), and the inflammatory caspase-1 (2, 3). Inflammasome assembly results in caspase-1 autocleavage and activation. Activated caspase-1 processes the proinflammatory cytokines pro-interleukin (IL)-1β and pro-IL-18 into their mature forms and cleaves gasdermin D (GSDMD) to release its active N terminus, which forms pores in the plasma membrane to induce cytokine release and pyroptotic cell death (4, 5, 6). To date, six inflammasomes have been well documented, including the nucleotide-binding oligomerization domain–like receptor (NLR) family members NLRP1, NLRP3, NLRP6, NLRC4, pyrin, and absent in melanoma 2 (AIM2) inflammasomes (2, 7). Among these inflammasomes, the NLRP3 inflammasome has been intensively investigated due to its protective role in host defense against various infections as well as its pathogenic role in several inflammatory disorders (8, 9).

The mechanism underlying NLRP3 inflammasome activation remains not fully understood. A diverse spectrum of stimuli, including ATP, pore-forming toxin (*e.g.*, nigericin), and particulate matter (*e.g.*, monosodium urate crystals, amyloids, and silica), has been shown to activate the NLRP3 inflammasome (10, 11, 12). Therefore, it is unlikely that NLRP3 directly recognizes these stimuli. Instead, molecular or cellular signal events downstream of these stimuli, such as altered intracellular ion homeostasis, mitochondrial dysfunction, reactive oxygen species production, and lysosomal damage, have been proposed to trigger NLRP3 inflammasome activation (10, 11, 12). Most NLRP3 stimuli trigger potassium efflux, which has been considered a critical signal for NLRP3 inflammasome activation (13, 14). However, how NLRP3 senses the drop in intracellular potassium concentration remains unknown. Furthermore, several NLRP3-interacting proteins, including heat-shock protein 90 (HSP90), SGT1, TXNIP, NEK7, DDX3X, and RACK1, have been shown to regulate NLRP3 inflammasome activation (15, 16, 17, 18, 19, 20). However, the molecular mechanism of these regulators remains to be further defined.

NLRP3 consists of three domains: an N-terminal pyrin domain (PYD), a central NAIP, CIITA, HET-E, and TP1 (NACHT) domain, and a C-terminal leucine-rich repeat (LRR) domain (21). NLRP3 interacts with the adaptor protein ASC through PYD-PYD interactions to induce the formation of filamentous aggregates known as ASC specks (22, 23). The NACHT domain has an adenosine triphosphatase activity required for NLRP3 conformational change and oligomerization (24, 25). In contrast, the role of the LRR domain in NLRP3 inflammasome activation is more complex. Earlier studies have suggested that the LRR domain has an autoinhibitory function through intramolecular interactions (26). Recently, several posttranslational modifications, including phosphorylation and ubiquitination, have been found in the LRR domain to regulate NLRP3 inflammasome activation (27, 28, 29, 30). However, there are conflicting reports on whether the entire LRR domain plays a role in NLRP3 inflammasome activation. Some studies have shown that the replacement of the LRR domain by the LacZ protein or its deletion abolished NLRP3 inflammasome activation (31, 32). Furthermore, structural studies have shown that the LRR domain mediated the NLRP3-NEK7 interaction and the formation of the NLRP3 cage structure, which disperses the *trans*-Golgi network at the early stage of the inflammasome pathway (33, 34). However, others have shown that the LRR domain is dispensable for NLRP3 inflammasome activation (35, 36).

In this study, we have used mouse knock-in (KI) macrophages that express an endogenous NLRP3 mutant lacking the LRR domain and NLRP3-deficient macrophages that are reconstituted with NLRP3 mutants lacking the LRR domain. Our results demonstrate that the LRR domain is required for NLRP3 inflammasome activation. Furthermore, our results show that the LRR domain mediates NLRP3 self-association, oligomerization, and interaction with its essential regulator NEK7.

## Results

### Deletion of the LRR domain from endogenous NLRP3 protein impairs nigericin-induced NLRP3 inflammasome activation in macrophages

**Figure 1:**
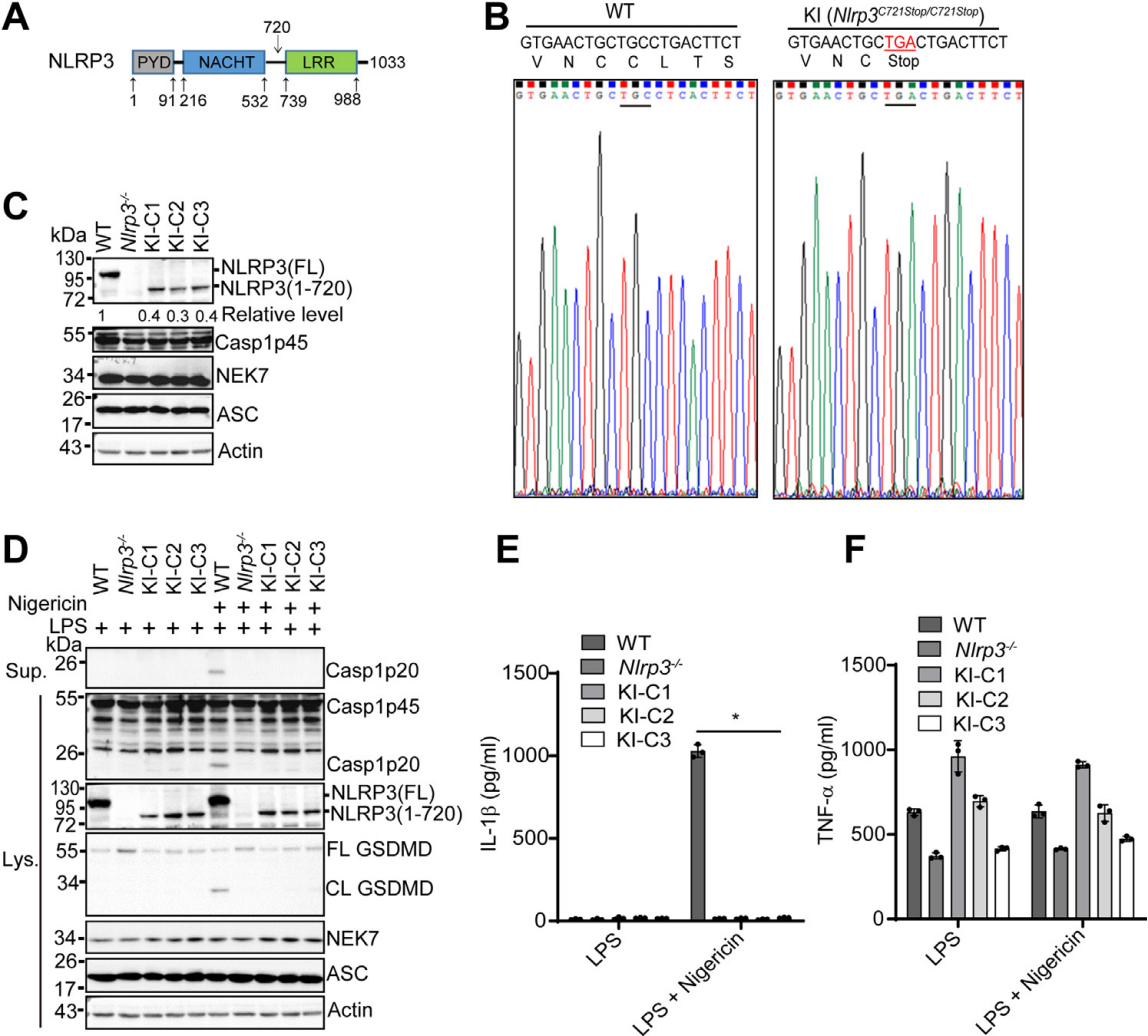
**The LRR domain is required for nigericin-induced endogenous NLRP3 inflammasome activation in macrophages.***A*, schematic presentation for domains of mouse NLRP3 (UniProtKB: Q8R4B8). The bottom numbers show the positions of amino acid residues at the start and end of each domain. The top number (720) shows the position of the last amino acid residue for an NLRP3 LRR deletion mutant [NLRP3 (1–720)]. *B*, sequencing verification for the replacement of a cysteine codon (TGC) by a stop codon (TGA) in mouse *Nlrp3* gene. *C*, immunoblot analysis of NLRP3 inflammasome components in wildtype (WT), *Nlrp3*^*−/−*^, and knock-in macrophages (three individual clones: KI-C1, KI-C2, KI-C3). Actin was used as a control. Representative blots (n = 2). The band density of NLRP3 was measured by ImageJ and normalized to the full-length NLRP3. *D*, macrophages were stimulated with LPS (4 h) alone or plus 5 μM nigericin (1 h). Cell lysates and supernatants were immunoblotted with indicated antibodies. Representative blots (n = 3). Measurement of IL-1β (*E*) and TNF-α (*F*) in the supernatants from stimulated macrophages by ELISA. Representative data(n = 3). ∗*p* < 0.05 (one-way ANOVA). Data are the mean ± SD of triplicate wells. CL, cleaved; FL, full-length; LPS, lipopolysaccharide; LRR, leucine-rich repeat.

Previous studies showed conflicting results on the role of the LRR domain in NLRP3 inflammasome activation. Notably, most of these studies overexpressed NLRP3 mutants lacking the LRR domain by transfection or viral infection or replaced the LRR domain of the endogenous NLRP3 protein with a large LacZ protein, which may interfere with NLRP3 function (31, 32, 35, 36). To determine the exact role of this LRR domain in NLRP3 inflammasome activation, we used the CRISPR-Cas9 gene-editing system to generate immortalized mouse bone marrow-derived macrophages (iBMDMs) that endogenously express an NLRP3 mutant lacking the LRR domain. An NLRP3 mutant [NLRP3(1–720)] was chosen as it lacks the entire LRR domain and has been reported to fully restore NLRP3 inflammasome activation in NLRP3-deficient mouse macrophages (Fig. 1*A*) (35). We selected three KI macrophage clones (*Nlrp3*^*C721Stop/C721Stop*^) in which a stop codon (TGA) has successfully replaced the cysteine (C721) codon (TGC) at the coding region of mouse *Nlrp3* gene (Fig. 1*B*). These KI macrophages are expected to express an NLRP3 mutant that consists of the first 720 amino acid residues and lacks the entire LRR domain.

We assessed the protein expression of endogenous NLRP3 protein from both wildtype (parent cells) and KI macrophages by Western blot. An antibody that recognized an epitope in the N-terminal pyrin domain of NLRP3 protein (Cryo-2) was used to detect both full-length and truncation mutant NLRP3 from these macrophages. In addition, macrophages derived from *Nlrp3*^*−/−*^ mice were included as controls in our experiments. The full-length NLRP3 (∼118 kDa) was detected in wildtype macrophages at the resting state (Fig. 1*C*). As expected, all three KI macrophage clones expressed the short version of NLRP3 protein (∼79 kDa), further confirming our sequencing results (Fig. 1*C*). Of note, no protein band appeared in the lane for *Nlrp3*^*−/−*^ macrophages, indicating the specificity of our antibody (Fig. 1*C*). Importantly, the detected protein level of this NLRP3 mutant from each clone was markedly lower (less than 50%) when compared with that of full-length NLRP3 expressed in wildtype macrophages (Fig. 1*C*). Additionally, the mRNA levels of this NLRP3 mutant in KI macrophages were also lower than those of the full-length NLRP3 in wildtype macrophages (Fig. S1*A*). However, the expression levels of other NLRP3 inflammasome components, including caspase-1, NEK7, and ASC, were comparable between wildtype and KI macrophages (Fig. 1*C*). Next, we assessed NLRP3 inflammasome activation in these macrophages under the conditions of lipopolysaccharide (LPS) priming or LPS priming plus nigericin treatment. Previous studies have suggested that the LRR domain keeps an inactive conformation of NLRP3 through intramolecular interaction (26). Similar to wild-type macrophages, KI macrophages did not exhibit caspase-1 activation, GSDMD cleavage, or IL-1β secretion after LPS priming (Fig. 1, *D* and *E*). Nigericin, a classic NLRP3 stimulus, induced NLRP3 inflammasome activation in wildtype macrophages, as revealed by caspase-1 activation, GSDMD cleavage, and IL-1β secretion (Fig. 1, *D* and *E*). In contrast, KI macrophages, similar to NLRP3-deficient macrophages, were defective in NLRP3 inflammasome activation after LPS priming plus nigericin treatment (Fig. 1, *D* and *E*). The secretion of TNF-α, an inflammasome-independent cytokine, was comparable among these macrophages (Fig. 1*F*). Collectively, these results indicate that the LRR domain is required for nigericin-induced NLRP3 inflammasome activation in macrophages.

### Deletion of the LRR domain inhibits NLRP3 inflammasome activation by diverse NLRP3 stimuli

**Figure 2:**
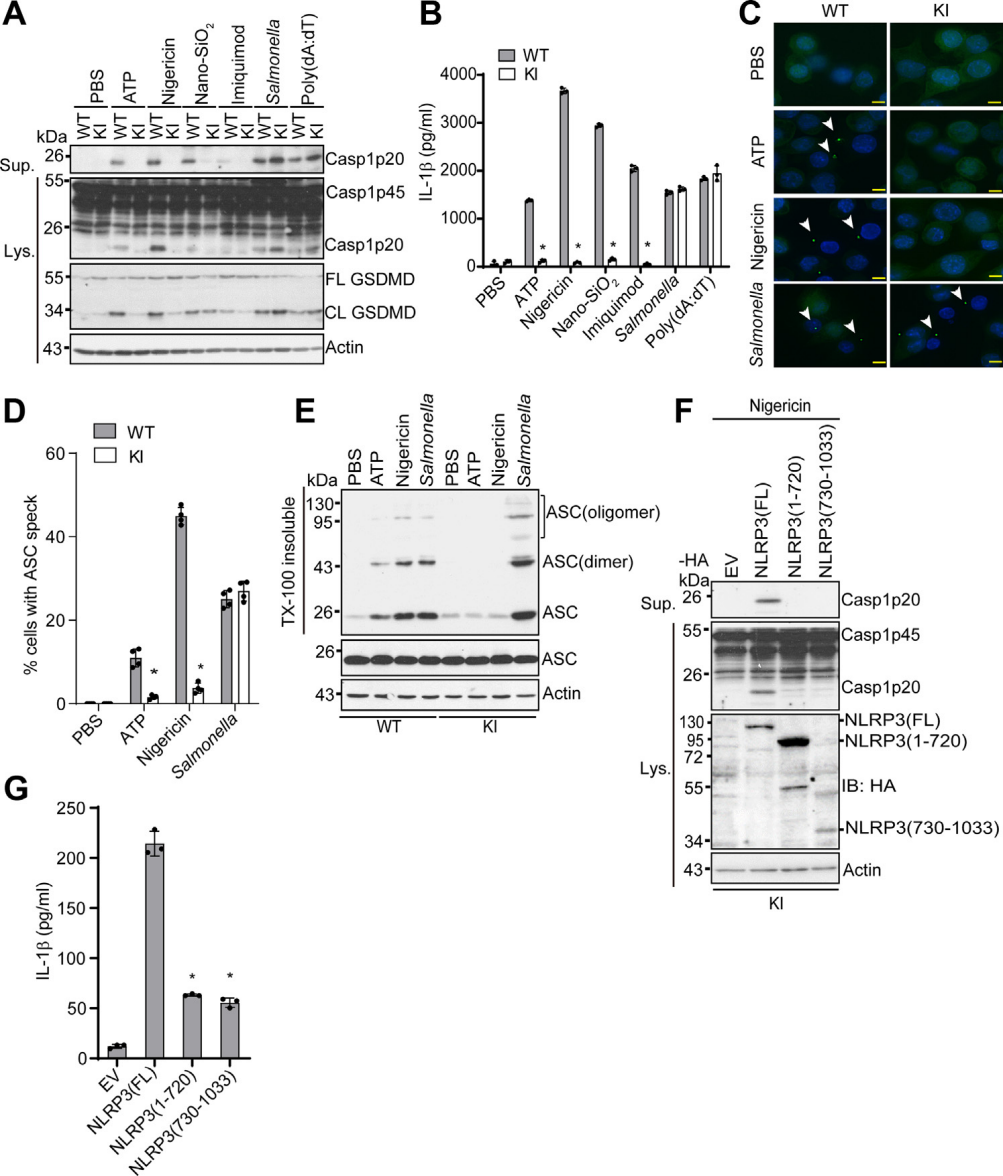
**The LRR domain is required for NLRP3 inflammasome activation induced by diverse stimuli.***A*, LPS-primed WT and NLRP3 1–720 knock-in (KI) macrophages were primed with LPS (4 h) and then stimulated with PBS (mock), 5 mM ATP (1 h), 5 μM nigericin (1 h), 200 μg/ml Nano-SiO_2_ (4 h), 15 μg/ml Imiquimod (4 h), 4 μg/ml poly(dA:dT) (4 h) or *Salmonella* (m.o.i= 10, 2 h). Cell lysates and supernatants were immunoblotted with indicated antibodies. Representative blots (n = 3). *B*, measurement of IL-β in the supernatants from (*A*) by ELISA. Representative data (n = 3). ∗*p* < 0.05 (unpaired two-sided *t* test). Data are the mean ± SD of triplicate wells. *C*, ASC immunostaining in wildtype or KI macrophages primed with LPS and stimulated with PBS, ATP, nigericin, or *Salmonella*. Scale bars, 10 μm. ASC specks are indicated by *white arrows*. Representative images (n = 3). *D*, quantification of ASC specks from (*C*). The percentage of ASC speck-containing cells was calculated from three different fields with at least 100 cells each. ∗*p* < 0.05 (unpaired two-sided *t* test). *E*, immunoblot analysis of ASC oligomerization from Triton X (TX)-100 insoluble and soluble fractions in WT and KI macrophages. *F*, KI macrophages were transduced with a lentiviral vector expressing C-terminal HA-tagged full-length (FL), LRR domain deletion mutant [NLRP3 (1–720)], or the LRR domain alone [NLRP3 (730–1033)]. Macrophages were primed with LPS and then stimulated with nigericin. Cell lysates and supernatants were immunoblotted with indicated antibodies. Actin was used as a control. Representative blots (n = 3). *G*, measurement of IL-β in the supernatants from (F) by ELISA. ∗*p* < 0.05 (unpaired two-sided *t* test). Data are the mean ± SD of triplicate wells. ASC, apoptosis-associated speck-like protein containing a caspase-activation and recruitment domain; CL, cleaved; FL, full-length; LPS, lipopolysaccharide; LRR, leucine-rich repeat.

NLRP3 inflammasome activation is induced by potassium efflux-dependent (*e.g.*, ATP, nigericin, Nano-SiO_2_) or independent stimuli (*e.g.*, imiquimod) (11, 12). Given our data showing that KI macrophages were defective in nigericin-induced NLRP3 inflammasome activation, we next examined whether this defect was common to other NLRP3 stimuli. As they secreted a similar level of TNF-α after LPS priming, the KI macrophage clone KI-C2 (KI henceforth) and wildtype macrophages were used for the following experiments (Fig. 1*F*). Macrophages were stimulated with ATP, nigericin, Nano-SiO_2_, or imiquimod after LPS priming. KI macrophages showed markedly reduced caspase-1 activation, GSDMD cleavage, and IL-1β secretion when compared to wildtype macrophages (Fig. 2, *A* and *B*). In contrast, KI macrophages had comparable AIM2 inflammasome activation induced by poly(dA:dT) or NLRC4 inflammasome activation by *Salmonella* when compared to wildtype macrophages (Fig. 2, *A* and *B*). Inflammasome activation induces the formation of ASC aggregates called ASC specks, which are composed of ASC oligomers in the cytosol (22). Both ATP and nigericin induced ASC speck formation in wildtype macrophages, whereas almost no ASC speck was detected in KI macrophages for both stimuli (Fig. 2, *C* and *D*). However, KI macrophages had comparable ASC speck formation in response to *Salmonella* as compared to wildtype macrophages (Fig. 2, *C* and *D*). Biochemical analysis revealed that KI macrophages had much less ASC oligomerization as compared to wildtype cells after treatment of ATP or nigericin, although they showed a comparable level of ASC oligomerization in response to *Salmonella* (Fig. 2*E*). To exclude whether other nonspecific factors besides this LRR domain deletion in endogenous NLRP3 attributed to the defect of NLRP3 inflammasome activation observed in KI macrophages, we expressed C-terminal HA-tagged NLRP3 full-length [NLRP3(FL)], LRR deletion mutant [NLRP3(1–720)], or the LRR domain [NLRP3(730–1033)] in KI macrophages by a lentiviral vector. Macrophage clones that expressed higher levels of the LRR deletion mutant protein than those of full-length NLRP3, indicated by anti-HA immunoblots, were selected for assessing nigericin-induced NLRP3 inflammasome activation (Fig. 2*F*). Expression of full-length NLRP3 restored NLRP3 inflammasome activation, indicated by caspase-1 activation and IL-1β secretion, in KI macrophages, while both the LRR deletion mutant and LRR domain alone failed to rescue NLRP3 inflammasome activation (Fig. 2, *F* and *G*). Taken together, these results indicated that the LRR domain is required for NLRP3 inflammasome activation in macrophages.

### NLRP3 mutants lacking the LRR domain fail to restore NLRP3 inflammasome activation in NLRP3-deficient macrophages

**Figure 3:**
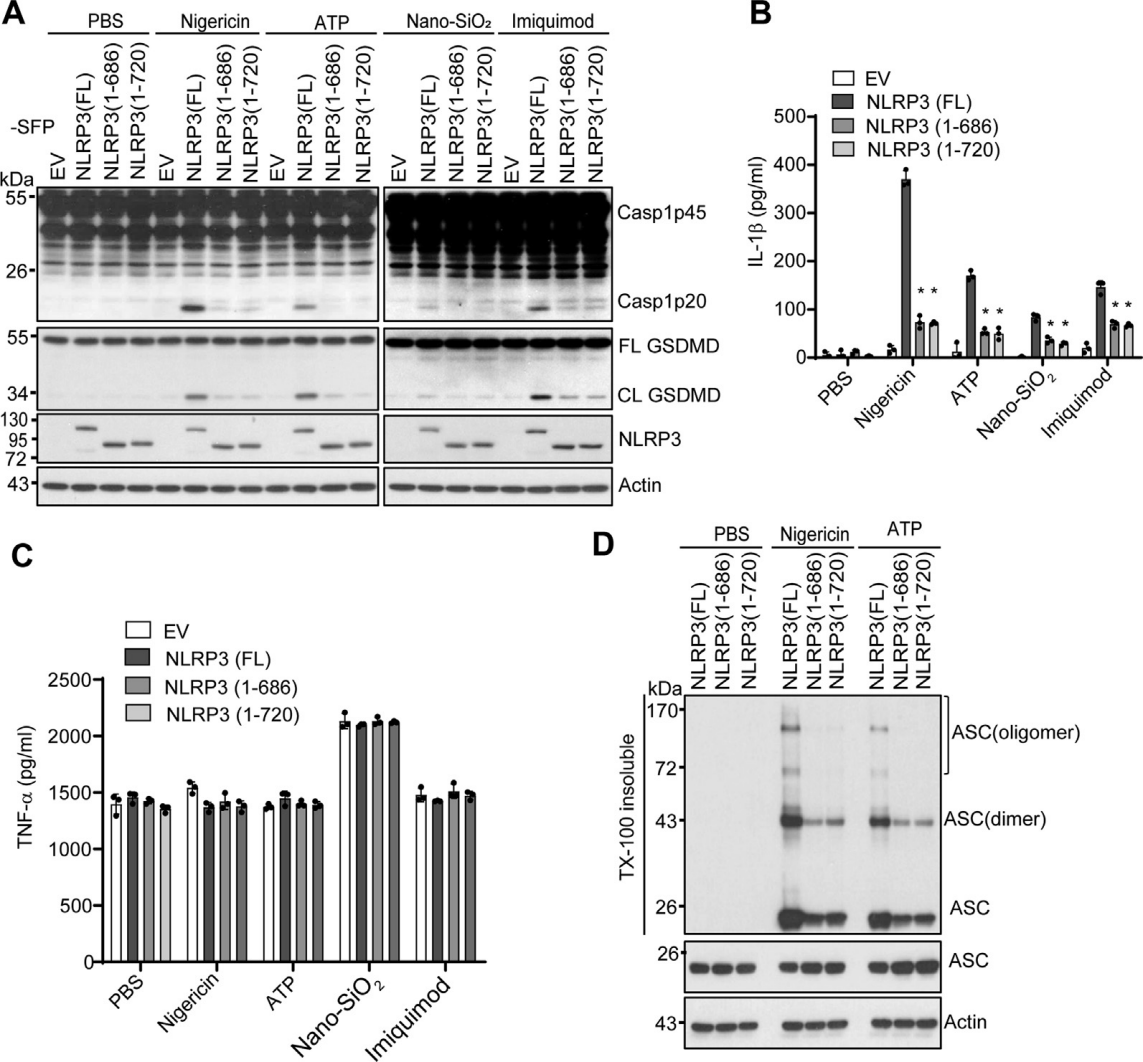
**LRR domain deletion mutants of NLRP3 fail to fully rescue NLRP3 inflammasome activation in mouse *Nlrp3***^***−/−***^**macrophages.***A*, mouse *Nlrp3*^*−/−*^ macrophages were transduced with a lentiviral vector expressing C-terminal SFP (S-tag, Flag, and streptavidin-binding tag)-tagged full-length (FL), LRR domain deletion mutant 1–686 [NLRP3 (1–686)], or LRR domain deletion mutant 1–720 [NLRP3 (1–720)]. LPS-primed macrophages were stimulated with PBS (mock), 5 μM nigericin (1 h), 5 mM ATP (1 h), 200 μg/ml Nano-SiO_2_ (4 h), or 15 μg/ml Imiquimod (4 h). Mixtures of cell lysates and supernatants were immunoblotted with indicated antibodies. Measurement of IL-β (*B*) and TNF-α (*C*) in the supernatants from stimulated macrophages by ELISA. *D*, immunoblot analysis of ASC oligomerization from Triton X (TX)-100 insoluble and soluble fractions in macrophages expressing indicated full-length or LRR deletion mutant of NLRP3. Representative blots (n = 3). Data are the mean ± SD of triplicate wells. ∗*p* < 0.05 (unpaired two-sided *t* test). ASC, apoptosis-associated speck-like protein containing a caspase-activation and recruitment domain; CL, cleaved; EV, empty vector. FL, full-length; LRR, leucine-rich repeat.

Several LRR domain-lacking mutants of NLRP3, including NLRP3 1–686 and 1–720, have been reported to fully restored inflammasome activation in mouse NLRP3-deficient macrophages (35). As shown in our data, the protein level of endogenously expressed protein NLRP3(1–720) in KI macrophages was lower than that of the full-length NLRP3 in macrophages. As a complementary approach, we constitutively expressed SFP (S-tag, Flag, and streptavidin-binding tag)-tagged full-length NLRP3, NLRP3 mutant 1–686, or 1–720 in mouse NLRP3-deficient macrophages and selected macrophage clones that expressed comparable protein levels of NLRP3 (Fig. 3*A*). In contrast to the previous study in which macrophages were primed with LPS for 11 h in the presence of doxycycline, we primed reconstituted macrophages with LPS for 4 h before stimulation with ATP, nigericin, nano-SiO_2_, or imiquimod (35). Consistent with our results from KI macrophages, macrophages reconstituted with NLRP3 mutant 1–686 or 1–720 showed a significant reduction in caspase-1 activation, GSDMD cleavage, and IL-1β secretion when compared to macrophages with full-length NLRP3 (Fig. 3, *A* and *B*). The levels of TNF-α secretion were comparable between macrophages reconstituted with wildtype or mutant NLRP3 under the same conditions (Fig. 3*C*). Accordingly, macrophages reconstituted with NLRP3 mutant 1–686 or 1–720 had less ASC oligomerization in response to ATP or nigericin when compared to macrophages with full-length NLRP3 (Fig. 3*D*). Furthermore, NLRP3-deficient macrophages that expressed an untagged NLRP3 mutant 1–720 through a constitutive or doxycycline-inducible system also failed to restore caspase-1 activation after nigericin stimulation (Fig. S1, *B* and *C*). Taken together, these results further indicated that the LRR domain is also essential for NLRP3 inflammasome activation in reconstituted NLRP3-deficient macrophages.

### Deletion of the LRR domain inhibits NLRP3 self-association and oligomerization

**Figure 4:**
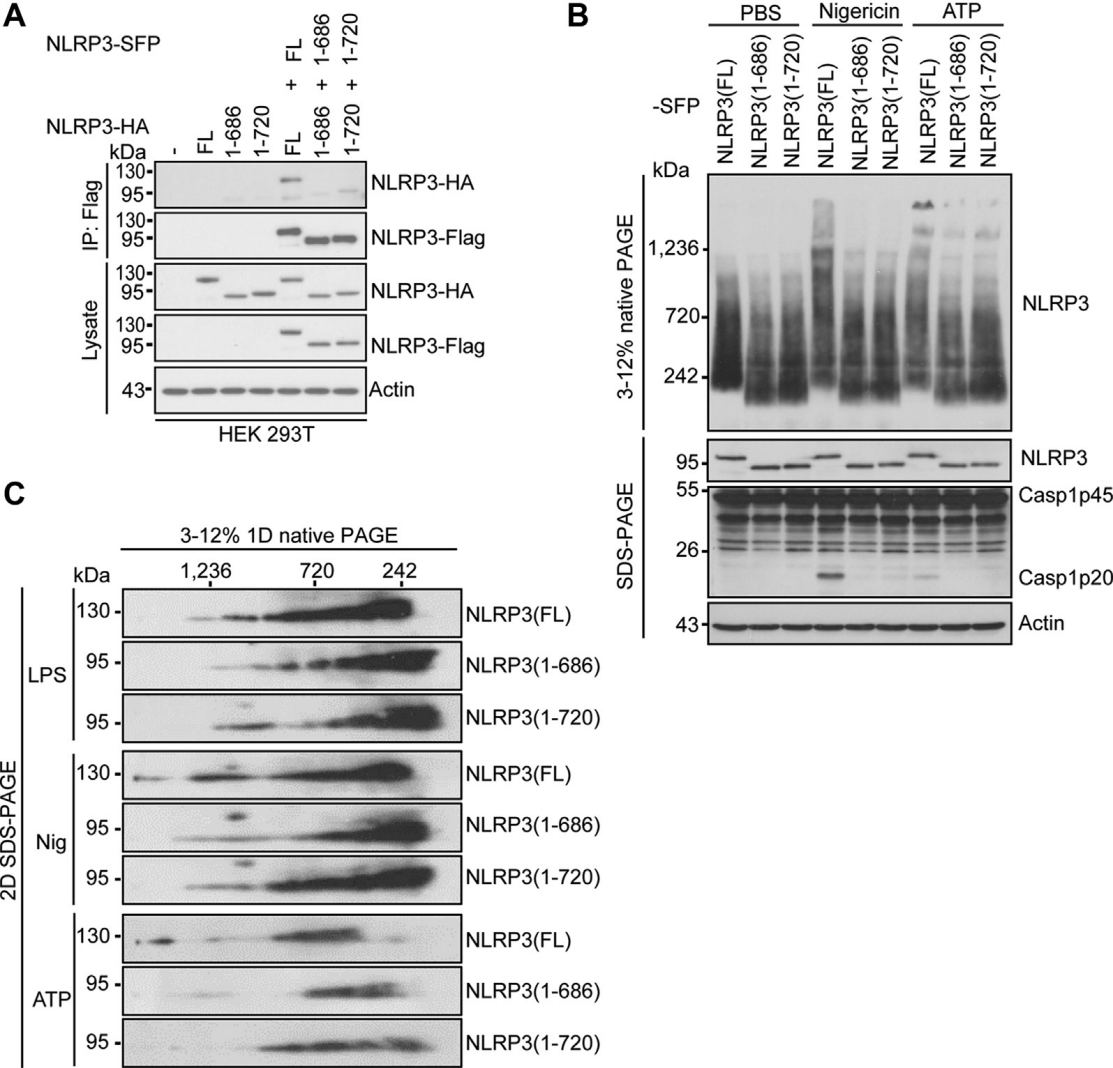
**LRR domain deletion mutants of NLRP3 show defects in NLRP3 self-association and oligomerization.***A*, HA-tagged mouse full-length NLRP3, LRR domain deletion mutant NLRP3 (1–686), or NLRP3 (1–720) was expressed alone or with indicated SFP-tagged protein in HEK 293T cells. Cell lysates were immunoprecipitated with an anti-Flag antibody and immunoblotted with indicated antibodies. *B*, mouse *Nlrp3*^*−/−*^ macrophages were reconstituted with SFP-tagged wildtype or mutant NLRP3 and stimulated with PBS (mock), ATP, or nigericin after LPS priming. NLRP3 oligomerization was analyzed by blue native PAGE and immunoblotting with an anti-Flag antibody. Cell lysates were also analyzed by SDS–PAGE and immunoblotting with indicated antibodies. *C*, macrophage cell lysates were separated by the blue native PAGE, followed by a second dimension of SDS–PAGE and Western blot. Representative blots (n = 3). FL, full-length; LRR, leucine-rich repeat; SFP, S-tag, Flag, and streptavidin-binding tag.

Recent structural studies with purified NLRP3 oligomers have revealed that NLRP3 self-associates through the LRR-LRR interaction (34, 37). To investigate how the LRR domain controls NLRP3 inflammasome activation, we assessed the effects of the LRR domain deletion on NLRP3 self-association and oligomerization in cells. We co-expressed SFP or HA-tagged full-length or NLRP3 mutants in HEK 293T cells. The full-length NLRP3 showed self-association as SFP-tagged NLRP3 pulled down HA-tagged NLRP3. In contrast, self-association for both NLRP3 mutants 1–686 and 1–720 was markedly reduced (Fig. 4*A*). In macrophages, after stimulation of NLRP3 stimuli, NLRP3 forms high-molecular-mass complexes, as indicated by protein native gels (17). We compared these high-molecular-mass forms of NLRP3 in cells reconstituted with full-length or the LRR deletion mutant. Macrophages reconstituted with the full-length NLRP3 displayed the high-molecular-mass NLRP3 complexes (>1200 kDa) after stimulation of ATP and nigericin. In contrast, these high-molecular-mass forms of NLRP3 were markedly reduced in macrophages reconstituted with NLRP3 truncation mutant 1–686 or 1–720 (Fig. 4*B*). Accordingly, in 2-D native gels, these high-molecular-mass forms of NLRP3 proteins were markedly reduced in macrophages reconstituted with NLRP3 mutant 1–686 or 1–720 as compared with the full-length NLRP3 (Fig. 4*C*). Taken together, our results indicate that the LRR domain mediates NLRP3 self-association and oligomerization.

### Deletion of the LRR domain abolishes the NEK7-NLRP3 interaction

**Figure 5:**
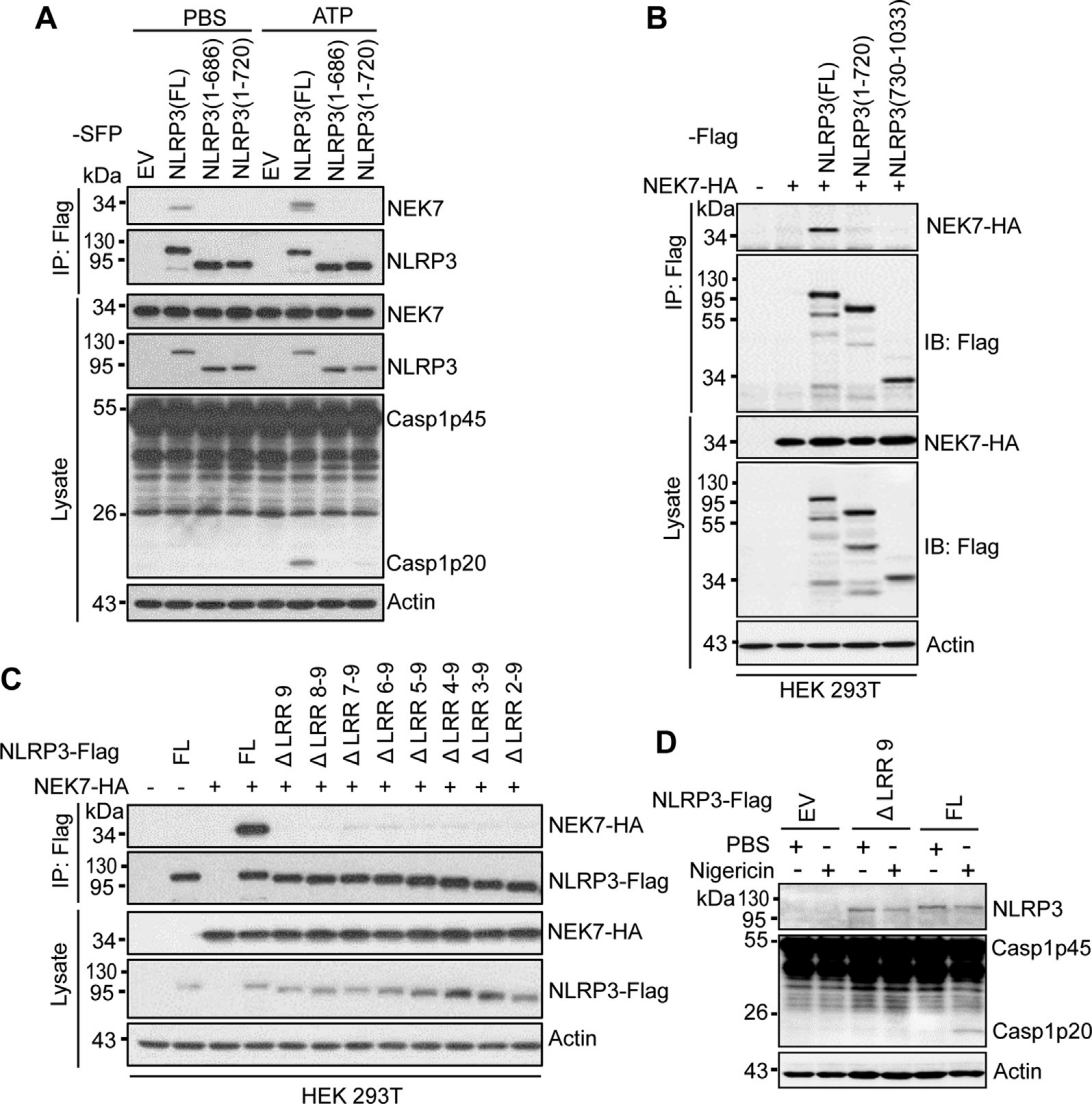
**Deletion of the LRR domain in NLRP3 abrogates NEK7-NLRP3 interaction.***A*, mouse *Nlrp3*^*−/−*^ macrophages were reconstituted with SFP-tagged full-length or mutant NLRP3 and stimulated with PBS (mock) or 5 mM ATP (1 h) after LPS priming. Cell lysates were immunoprecipitated with anti-Flag antibody and immunoblotted with indicated antibodies. *B*, Flag-tagged full-length, NLRP3 (1–720), or NLRP3 (730–1033) was co-expressed with HA-tagged NEK7 in HEK 293T cells. Cell lysates were immunoprecipitated with an anti-Flag antibody and immunoblotted with indicated antibodies. *C*, Flag-tagged NLRP3 mutants with deletion of one or multiple leucine-repeats were co-expressed with HA-tagged NEK7 in HEK 293T cells. Cell lysates were immunoprecipitated with an anti-Flag antibody and immunoblotted with indicated antibodies. ΔLRR 9, NLRP3 (1–964); ΔLRR 8–9, NLRP3 (1–936); ΔLRR 7–9, NLRP3 (1–907); ΔLRR 6–9, NLRP3 (1–879); ΔLRR 5–9, NLRP3 (1–850); ΔLRR 4–9, NLRP3 (1–818); ΔLRR 3–9, NLRP3 (1–793); ΔLRR 2–9, NLRP3 (1–765). *D*, mouse *Nlrp3*^*−/−*^ macrophages were reconstituted with Flag-tagged full-length or mutant NLRP3 (ΔLRR 9) and stimulated with PBS (mock) or 5 μM nigericin (1 h) after LPS priming. Mixtures of cell lysates and supernatants were immunoblotted with indicated antibodies. Representative blots (n = 3). EV, empty vector. FL, full-length; LPS, lipopolysaccharide; LRR, leucine-rich repeat; SFP, S-tag, Flag, and streptavidin-binding tag.

Previous studies have shown that the LRR domain in NLRP3 is required for the NEK7-NLRP3 interaction in HEK 293T cells (17, 18). Furthermore, a structural study of the NEK7-NLRP3 complex has shown that NEK7 interacts with the LRR domain of NLRP3 (33). Surprisingly, NLRP3 mutants lacking the LRR domain including 1–686 were reported to pull down NEK7 in macrophages (35). Therefore, we examined whether the LRR domain is required for NLRP3-NEK7 interaction in our reconstituted macrophages. Both NLRP3 mutants 1–686 and 1–720 failed to pull down NEK7 from macrophages stimulated with LPS or LPS plus ATP (Fig. 5*A*). The NLRP3 mutant 1–720 or the LRR domain alone also failed to pull down NEK7 from HEK 293T cells (Fig. 5*B*). According to the Uniprot/Swiss-Prot, there are nine LRRs in the LRR domain of NLRP3. To further define the exact LRR(s) of the LRR domain that is essential for NLRP3-NEK7 interaction, we generated a series of NLRP3 mutants which progressively lack one LRR starting from the C terminus. Co-expression of the Flag-tagged NLRP3 mutant and HA-tagged NEK7 in HEK 293T cells was used to assess the interaction between NEK7 and NLRP3 mutants. All of these NLRP3 mutants that lack one or several LRRs in the LRR domain lost the ability to bind NEK7 (Fig. 5*C*). Furthermore, the NLRP3 mutant lacking one LRR (Δ LRR 9) failed to restore nigericin-induced caspase-1 activation in reconstituted NLRP3-deficient macrophages (Fig. 5*D*). Collectively, these results indicate that the intact LRR domain is essential for NEK7-NLRP3 interaction and NLRP3 inflammasome activation.

## Discussion

NLRP3 activation triggers the assembly of an inflammasome that plays a critical role in innate immunity. In addition to its N-terminal pyrin domain and central NACHT domain, NLRP3 contains an LRR domain at its C terminus. Several posttranslational modifications have been found on the LRR domain to regulate NLRP3 inflammasome activation (11, 12). In this study, we investigated the role of this LRR domain in NLRP3 inflammasome activation in mouse macrophages. Our results show that the LRR domain is required for NLRP3 inflammasome activation in macrophages. Furthermore, we show that the LRR domain mediates NLRP3 self-association, oligomerization, and interaction with NEK7, which are critical molecular events for NLRP3 inflammasome activation.

Our results showed that the protein level of NLRP3 1–720 in KI macrophages was much less than that of full-length NLRP3 in wildtype cells. This reduction might result from the nonsense-mediated decay of NLRP3 1–720 mRNA after the introduction of the stop codon in the *Nlrp3* gene (Fig. S1*A*) (38). However, our reconstitution experiments showed that the protein level of this NLRP3 LRR deletion mutant (without a tag) was consistently lower than that of the full-length NLRP3, suggesting that the LRR domain promotes NLRP3 stability in macrophages (Fig. S1, *B* and *C*). This role of the LRR domain in NLRP3 protein stability, although it has not been explored further, has been suggested in previous studies (31). In addition, previous studies report that the chaperone HSP90 interacts with NLRP3 *via* the LRR domain and stabilizes the NLRP3 protein (15). Therefore, we hypothesize that the deletion of the LRR domain in NLRP3 might cause the loss of this HSP90-mediated protection and thus render its degradation by the proteasome. In support of this hypothesis, the E3 ubiquitin ligase Cbl-b has been reported to ubiquitinate the NACHT domain at K496 for proteasome-mediated degradation of NLRP3 (28). Interestingly, short NLRP3 transcripts that encode NLRP3 LRR domain truncation forms have been reported in mouse and human cells (39, 40). It remains unknown whether those truncation proteins of NLRP3 undergo degradation in cells.

There are conflicting reports on the role of the LRR domain in NLRP3 inflammasome activation. Previous studies have suggested that the LRR domain plays an inhibitory role in NLRP3 inflammasome activation (26). However, we did not observe constitutive activation of the NLRP3 inflammasome in mouse macrophages expressing an endogenous or exogenous LRR domain-lacking NLRP3 mutant. Recently, Hafner-Bratkovič *et al.*(35) reported that mouse NLRP3 truncation mutants including 1–686 and 1–720 fully restored NLRP3 inflammasome activation in NLRP3-deficient macrophages, suggesting a dispensable role for this LRR domain in NLRP3 inflammasome activation. In contrast, Niu *et al.*(32) reported that an NLRP3 mutant 1–688 (mouse 1–686) failed to restore NLRP3 inflammasome activation in NLRP3-deficient human monocytic U937 cells. In addition, BMDMs expressing an NLRP3 mutant, in which the LRR domain was replaced by a LacZ protein, were defective in NLRP3 inflammasome activation induced by monosodium urate crystals (31). Consistent with the latter two studies, our results indicate that the LRR domain is required for NLRP3 inflammasome activation in mouse macrophages. In our KI macrophages that express an endogenous NLRP3 mutant lacking the LRR domain, NLRP3 inflammasome activation was completely abrogated in response to all tested NLRP3 stimuli. This defect may also partially be attributed to the low protein level of this endogenous NLRP3 mutant in KI macrophages. However, overexpression of this NLRP3 mutant failed to restore NLRP3 inflammasome activation in these KI macrophages, suggesting an intrinsic role for the LRR domain in NLRP3 inflammasome activation. In addition, macrophages expressing NLRP3 truncation mutant 1–686 or 1–720, to a similar level as the full-length protein, were defective in NLRP3 inflammasome activation in our reconstitution experiments. We do not know the exact reasons for the above disparities. One possibility is that Hafner-Bratkovič *et al.*(35) induced a high level of NLRP3 proteins by using a longer time (11 h) of LPS priming in a doxycycline-inducible system. This experimental setting might induce a high level of protein expression and thus bypass the requirement of the LRR domain in NLRP3 inflammasome activation in macrophages. This is likely also applied to the reconstituted NLRP3 inflammasome in HEK 293 T cells (36). However, we failed to reproduce these results in macrophages using a similar doxycycline-inducible system (Fig. S1*C*). Importantly, we found that LRR deletion reduced NLRP3 self-association and oligomerization. Consistent with this observation, NLRP3 mutants with mutations in the LRR domain have been reported to be defective in the formation of a double-ring cage structure of mouse NLRP3 (34). Therefore, it is conceivable that deletion of the LRR will abolish the NLRP3 cage formation and subsequent inflammasome activation. Additionally, we further confirm and extend previous findings that an intact LRR domain in NLRP3 is required for its interaction with NEK7 (17, 18). Since the NLRP3 cage structure is not compatible with NEK7 binding, it remains to be determined when and where the LRR domain is involved in the interaction with NEK7 in this inflammasome activation pathway.

In summary, our findings demonstrated a critical role for the LRR domain in NLRP3 inflammasome activation in macrophages. Our results reveal that the LRR domain controls inflammasome activation by mediating NLRP3 self-association, oligomerization, and interaction with NEK7.

## Experimental procedures

### Antibodies and reagents

Anti-Flag (A00187–200), anti-HA (A01244–100), anti-Actin (A00730–100), and protein G resin (L00209) were purchased from GenScript. Anti-NLRP3 (AG-20B-0014-C100) was purchased from Adipogen. Anti-GSDMD (ab209845) and anti-NEK7 (ab133514) were purchased from Abcam. Anti-ASC (67824) was purchased from CST. Anti-mouse caspase-1 was a kind gift from Dr Gabriel Núñez (University of Michigan Medical School). Ultra-pure LPS (tlrl-pb5lps), Nano-SiO2 (tlrl-sio), and poly(dA:dT)/lyovec (tlrl-patc) were purchased from InvivoGen. Nigericin (481990), doxycycline hydrochloride (10592–13–9), and EDTA-free protease inhibitor cocktail (11873580001) were purchased from Sigma. *Salmonella* strain SL1344 was originally from Dr Denise Monack (Stanford University). The constructs for NLRP3-Flag, NLRP3-SFP, and NEK7-HA have been previously described (17).

### Cell culture

iBMDMs from WT C57BL/6 or *Nlrp3*^*−/−*^ mice were generated by murine retrovirus infection as previously described (41). iBMDMs were maintained in Iscove's Modified Dulbecco's medium (IMDM) (Thermo Fisher, 12440053) supplemented with 10% (vol/vol) FBS, L-glutamine, sodium pyruvate, and antibiotics (penicillin/streptomycin). For experiments, iBMDMs were seeded overnight into plates in IMDM with 1% (vol/vol) FBS. HEK 293 T cells were cultured in Dulbecco's modified Eagle's medium (Thermo Fisher, 11960044) supplemented with 10% (vol/vol) FBS, L-glutamine, sodium pyruvate, and antibiotics (penicillin/streptomycin). Cultured cells were tested to be free of *Mycoplasma* contamination.

### Generation of mouse Nlrp3^C721Stop/C721Stop^ KI macrophages

Alt-R CRISPR-Cas9 crRNA for mouse *Nlrp3* (targeting sequence: CACGGCAGAAGCTAGAAGTGAGG), tracrRNA (1072533), HDR donor oligos (Sense: TTCTGGCCTCCTCCTTTGCCATTCTAGACTGGTGAACTGCTGACTGACTTCTAGCTTCTGCCGTGGTCTCTTCTCAAGTCTAAGCACC; Antisense: GGTGCTTAGACTTGAGAAGAGACCACGGCAGAAGCTAGAAGTCAGTCAGCAGTTCACCAGTCTAGAATGGCAAAGGAGGAGGCCAGAA), Cas9 nuclease (1081059), and HDR Enhancer (1081072) were purchased from IDT, and the ribonucleoprotein complexes were prepared according to the manufacturer’s protocol. The ribonucleoprotein complexes were delivered into iBMDMs by electroporation with nucleofector kit V (Lonza, VCA-1003). Cell clones harboring *Nlrp3*^*C721Stop*^ homologous alleles were identified by sequencing.

### Inflammasome activation assay

iBMDMs were seeded overnight into 12-well plates at a density of 5 × 10^5^ per well in IMDM containing 1% (vol/vol) FBS. Cells were primed with 200 ng ml^−1^ ultrapure LPS for 4 h in serum-free IMDM. After priming, cells were then stimulated with ATP (5 mM, 1 h), nigericin (5 μM, 1 h), Nano-SiO_2_ (200 μg ml^−1^, 4 h), imiquimod (20 μg ml^−1^, 4 h), poly(dA:dT) (2 μg ml^−1^, 4 h), or *Salmonella* [multiplicity of infection (m.o.i.) = 10, 2 h]. For the doxycycline-inducible system, macrophages were treated with LPS (100 ng ml^−1^) and doxycycline (1 μg ml^−1^) for 12 h before stimulation with nigericin (5 μM, 1 h). After stimulation, culture supernatants were collected and cells were directly lysed with 150 μl of 2 × Laemmli buffer (Bio-Rad, 1610737). Proteins in culture supernatants, cell lysates, or mixtures of cell lysates and supernatants, were separated by SDS-PAGE and transferred onto PVDF membranes by using the Trans-Blot Turbo system (Bio-Rad). Membranes were immunoblotted with anti-caspase-1, anti-GSDMD, or other indicated antibodies. When specified, equal amounts of cell lysates and supernatants were combined for immunoblotting. The immunoblotting images were developed with HyBlot ES high sensitivity autoradiography films (Denville Scientific, E3218) or captured digitally by using ChemiDoc Imaging System (Bio-Rad). The release of cytokines IL-1β and TNF-α into supernatants was determined with ELISA kits (R&D Systems, DY401–05 and DY410–05) according to the manufacturer’s instructions.

### cDNA synthesis and real-time RT-PCR

iBMDMs were stimulated with LPS (200 ng ml^−1^) for 4 h or left unstimulated. Total RNA was extracted from iBMDMs using E.Z.N.A. Total RNA kit I (Omega Bio-Tek, R6834–01) according to the manufacturer’s instructions. cDNA was synthesized using a High-Capacity-RNA-to-cDNA kit (Fisher Scientific, 43–874–06). Real-time PCR was performed using SYBR Green Master Mix (Fisher Scientific, 43–444–63) at a 7500 Real-Time PCR system. The primers sequence was: Nlrp3 forward, CCCTTGGAGACACAGGACTC; Nlrp3 reverse, GAGGCTGCAGTTGTCTAATTCC; GAPDH forward, AGCTTGTCATCAACGGGAAG; GAPDH reverse, TTTGATGTTAGTGGGGTCTCG; The relative expression level of Nlrp3 to GAPDH was calculated using the 2^(−ct)^ method and normalized to the level of unstimulated wildtype iBMDMs.

### Reconstitution of the full-length NLRP3 and its truncation mutants in iBMDMs

SFP-tagged, HA-tagged, or untagged full-length mouse NLRP3 or its truncation mutant [NLRP3(1–686), NLRP3(1–720), or NLRP3(730–1033)] were cloned into lentiviral plasmid pHIV-EGFP (Addgene, 21373) or pINDUCER21 (Addgene, 46948). Each lentiviral construct was co-transfected with package plasmids pCMV-VSV-G (Addgene, 8454) and pCMV-dR8.2 dvpr (Addgene, 8455) into HEK 293T cells for 96 h. Lentiviruses in the culture supernatants were concentrated by using lenti-X concentrator (Takara Bio, 631231). iBMDMs were seeded into 6-well plates overnight at a density of 1 × 10^5^ per well. Cells were then transduced with lentiviruses in the presence of 8 μg ml^−1^ polybrene (Sigma, H9268) for 24 h before replacement with the fresh culture medium. After 3 to 4 days, transduced cells were sorted by flow cytometry using GFP as a marker. The expression of reconstituted proteins was determined by immunoblotting with an anti-HA, anti-NLRP3, or anti-Flag antibody. Macrophages expressing comparable levels of GFP were used in bulk for experiments with untagged NLRP3 proteins. For experiments with tagged NLRP3 proteins, cell clones of transduced macrophages that expressed different levels of NLRP3 proteins were selected by serial dilution and western blots.

### Transfection, immunoprecipitation, and pull-down assay

HEK 293T cells were seeded into 6-well plates overnight at a density of 6.25 × 10^5^ cells per well. Plasmids expressing HA-tagged NEK7, Flag, or SFP-tagged wild-type NLRP3, or its truncation mutant were single- or co-transfected into HEK 293T cells by Lipofectamine LTX (ThermoFisher, 15338100) for 16 h. After transfection, cells were washed with ice-cold PBS twice and lysed in ice-cold lysis buffer [50 mM Tris-HCl (pH 7.4), 2 mM EDTA, 150 mM NaCl, 0.5% (vol/vol) Nonidet P-40, 1 × EDTA-free protease inhibitor cocktail) at 4 °C for 10 min. Cell debris was removed by centrifugation at 12,000*g* for 10 min at 4 °C. Cell lysates were precleared by incubation with protein G agarose beads for 1 h at 4 °C. Precleared lysates were then incubated with an anti-Flag (1:200) antibody at 4 °C overnight. The proteins bound by the antibody were pulled down with protein G beads and subjected to immunoblotting analysis.

### ASC speck staining and imaging

iBMDMs were seeded overnight into an 8-well permanox chamber slide at a density of 1 × 10^5^ cells per well. Cells were primed with 200 ng ml^−1^ LPS for 4 h in serum-free IMDM and then stimulated with 5 mM ATP (30 min), 5 μM nigericin (1 h), or *Salmonella* (m.o.i = 10, 4 h). After stimulation, cells were washed once with PBS and then fixed in 4% paraformaldehyde for 20 min at room temperature. Fixed cells were permeabilized with 0.2% Triton X-100 for 5 min and blocked with PBS buffer containing 5% BSA for 1 h. Cells were incubated overnight with an anti-ASC antibody (1:200) in the blocking buffer. After washing three times with PBS, cells were incubated for 1 h with Alexa-Fluor-488 conjugated anti-rabbit secondary antibody (1:5000) in the blocking buffer. Cells were washed with PBS three times and mounted with Prolong Diamond Antifade Mountant with DAPI (Thermo Fisher, P36965). Cell images were taken using a Nikon E-800 microscope system and processed with ImageJ.

### ASC oligomerization assay

iBMDMs were seeded overnight in 6-well plates at a density of 1 × 10^6^ cells per well. Cells were primed with 200 ng ml^−1^ LPS for 4 h in serum-free IMDM and then stimulated with 5 mM ATP (30 min), 5 μM nigericin (1 h), or *Salmonella* (m.o.i = 10, 4 h). After removing the medium, cells were directly lysed in wells for 15 min at 4 °C with 300 μl of ice-cold PBS buffer supplemented with 0.5% Triton X-100, 0.5 mM PMSF, and 1 × EDTA-free protease inhibitor cocktail. Cell lysates were collected by scrapping from each well and transferred to Eppendorf tubes. Lysates were separated into supernatants (TritonX-100-soluble fraction) and pellet (Triton X-100-insoluble fraction) by centrifugation at 6000*g* for 15 min at 4 °C. The Triton X-100-insoluble fractions were washed with PBS twice and cross-linked for 30 min at room temperature with 2 mM bis[sulfosuccinimidy] suberate (BS^3^) (Thermo Fisher, 21580). The cross-linked pellets were spun down at 6000*g* for 15 min and dissolved in an SDS sample buffer for subsequent immunoblotting analysis.

### Blue native PAGE and 2D PAGE

Blue native gel electrophoresis was performed as previously described (42). Briefly, iBMDMs were seeded overnight into 6-well plates at a density of 1 × 10^6^ cells per well and stimulated as indicated. After stimulation, cells were washed once with cold PBS and then lysed in ice-cold native lysis buffer (20 mM Bis-tris, 500 mM ε-aminocaproic acid, 20 mM NaCl, 10% (w/v) glycerol, 0.5% digitonin, 0.5 mM Na_3_VO_4_, 1 mM PMSF, 0.5 mM NaF, 1 × EDTA-free Roche protease inhibitor cocktail, pH 7.0) for 15 min on ice. Cell lysates were clarified by centrifugation at 20,000*g* for 30 min at 4 °C; proteins were separated in 4 to 12% blue native PAGE and then analyzed by Western blot. For the two-dimensional (2D) PAGE, the natively resolved gel slice was loaded into the well of 4 to 12% SDS–PAGE gel after being soaked in 1 × Laemmli buffer as previously described (42).

### Statistical analysis

Data are represented as mean ± SD. Statistical analysis was performed using unpaired two-tailed Student’s *t* test or one-way ANOVA with GraphPad 9.0. A *p* value less than 0.05 was considered statistically significant.

## Data availability

All data generated for this study are included in this article.

## Supporting information

This article contains Supporting information.

## Conflict of interest

The authors declare that they have no conflicts of interest with the contents of this article.

## References

[1] Schroder K., Tschopp J. (2010). The inflammasomes. Cell.

[2] Broz P., Dixit V.M. (2016). Inflammasomes: mechanism of assembly, regulation and signalling. Nat. Rev. Immunol..

[3] Rathinam V.A., Vanaja S.K., Fitzgerald K.A. (2012). Regulation of inflammasome signaling. Nat. Immunol..

[4] Martinon F., Burns K., Tschopp J. (2002). The inflammasome: a molecular platform triggering activation of inflammatory caspases and processing of proIL-beta. Mol. Cell..

[5] Shi J., Zhao Y., Wang K., Shi X., Wang Y., Huang H. (2015). Cleavage of GSDMD by inflammatory caspases determines pyroptotic cell death. Nature.

[6] He W.T., Wan H., Hu L., Chen P., Wang X., Huang Z. (2015). Gasdermin D is an executor of pyroptosis and required for interleukin-1beta secretion. Cell Res..

[7] Sharma D., Kanneganti T.D. (2016). The cell biology of inflammasomes: mechanisms of inflammasome activation and regulation. J. Cell Biol..

[8] Anand P.K., Malireddi R.K., Kanneganti T.D. (2011). Role of the nlrp3 inflammasome in microbial infection. Front Microbiol..

[9] Guo H., Callaway J.B., Ting J.P. (2015). Inflammasomes: mechanism of action, role in disease, and therapeutics. Nat. Med..

[10] Sutterwala F.S., Haasken S., Cassel S.L. (2014). Mechanism of NLRP3 inflammasome activation. Ann. N. Y. Acad. Sci..

[11] Kelley N., Jeltema D., Duan Y., He Y. (2019). The NLRP3 inflammasome: an overview of mechanisms of activation and regulation. Int. J. Mol. Sci..

[12] Swanson K.V., Deng M., Ting J.P. (2019). The NLRP3 inflammasome: molecular activation and regulation to therapeutics. Nat. Rev. Immunol..

[13] Munoz-Planillo R., Kuffa P., Martinez-Colon G., Smith B.L., Rajendiran T.M., Nunez G. (2013). K(+) efflux is the common trigger of NLRP3 inflammasome activation by bacterial toxins and particulate matter. Immunity.

[14] Petrilli V., Papin S., Dostert C., Mayor A., Martinon F., Tschopp J. (2007). Activation of the NALP3 inflammasome is triggered by low intracellular potassium concentration. Cell Death Differ..

[15] Mayor A., Martinon F., De Smedt T., Petrilli V., Tschopp J. (2007). A crucial function of SGT1 and HSP90 in inflammasome activity links mammalian and plant innate immune responses. Nat. Immunol..

[16] Zhou R.B., Tardivel A., Thorens B., Choi I., Tschopp J. (2010). Thioredoxin-interacting protein links oxidative stress to inflammasome activation. Nat. Immunol..

[17] He Y., Zeng M.Y., Yang D., Motro B., Nunez G. (2016). NEK7 is an essential mediator of NLRP3 activation downstream of potassium efflux. Nature.

[18] Shi H., Wang Y., Li X., Zhan X., Tang M., Fina M. (2015). NLRP3 activation and mitosis are mutually exclusive events coordinated by NEK7, a new inflammasome component. Nat Immunol..

[19] Duan Y., Zhang L., Angosto-Bazarra D., Pelegrin P., Nunez G., He Y. (2020). RACK1 mediates NLRP3 inflammasome activation by promoting NLRP3 active conformation and inflammasome assembly. Cell Rep..

[20] Samir P., Kesavardhana S., Patmore D.M., Gingras S., Malireddi R.K.S., Karki R. (2019). DDX3X acts as a live-or-die checkpoint in stressed cells by regulating NLRP3 inflammasome. Nature.

[21] Wang L., Sharif H., Vora S.M., Zheng Y., Wu H. (2021). Structures and functions of the inflammasome engine. J. Allergy Clin. Immunol..

[22] Fernandes-Alnemri T., Wu J., Yu J.W., Datta P., Miller B., Jankowski W. (2007). The pyroptosome: A supramolecular assembly of ASC dimers mediating inflammatory cell death via caspase-1 activation. Cell Death Differ..

[23] Lu A., Magupalli V.G., Ruan J., Yin Q., Atianand M.K., Vos M.R. (2014). Unified polymerization mechanism for the assembly of ASC-dependent inflammasomes. Cell.

[24] Duncan J.A., Bergstralht D.T., Wang Y.H., Willingham S.B., Ye Z.M., Zimmermann A.G. (2007). Cryopyrin/NALP3 binds ATP/dATP, is an ATPase, and requires ATP binding to mediate inflammatory signaling. Proc. Natl. Acad. Sci. U. S. A..

[25] Sandall C.F., Ziehr B.K., MacDonald J.A. (2020). ATP-binding and Hydrolysis in inflammasome activation. Molecules.

[26] Dowds T.A., Masumoto J., Zhu L., Inohara N., Nunez G. (2004). Cryopyrin-induced interleukin 1beta secretion in monocytic cells: enhanced activity of disease-associated mutants and requirement for ASC. J. Biol. Chem..

[27] Py B.F., Kim M.S., Vakifahmetoglu-Norberg H., Yuan J. (2013). Deubiquitination of NLRP3 by BRCC3 critically regulates inflammasome activity. Mol. Cell..

[28] Tang J., Tu S., Lin G., Guo H., Yan C., Liu Q. (2020). Sequential ubiquitination of NLRP3 by RNF125 and Cbl-b limits inflammasome activation and endotoxemia. J. Exp. Med..

[29] Spalinger M.R., Kasper S., Gottier C., Lang S., Atrott K., Vavricka S.R. (2016). NLRP3 tyrosine phosphorylation is controlled by protein tyrosine phosphatase PTPN22. J. Clin. Invest..

[30] Tang J., Xiao Y., Lin G., Guo H., Deng H.X., Tu S. (2021). Tyrosine phosphorylation of NLRP3 by the Src family kinase Lyn suppresses the activity of the NLRP3 inflammasome. Sci. Signal..

[31] Hoffman H.M., Scott P., Mueller J.L., Misaghi A., Stevens S., Yancopoulos G.D. (2010). Role of the leucine-rich repeat domain of cryopyrin/NALP3 in monosodium urate crystal-induced inflammation in mice. Arthritis Rheum..

[32] Niu T., De Rosny C., Chautard S., Rey A., Patoli D., Groslambert M. (2021). NLRP3 phosphorylation in its LRR domain critically regulates inflammasome assembly. Nat. Commun..

[33] Sharif H., Wang L., Wang W.L., Magupalli V.G., Andreeva L., Qiao Q. (2019). Structural mechanism for NEK7-licensed activation of NLRP3 inflammasome. Nature.

[34] Andreeva L., David L., Rawson S., Shen C., Pasricha T., Pelegrin P. (2021). NLRP3 cages revealed by full-length mouse NLRP3 structure control pathway activation. Cell.

[35] Hafner-Bratkovic I., Susjan P., Lainscek D., Tapia-Abellan A., Cerovic K., Kadunc L. (2018). NLRP3 lacking the leucine-rich repeat domain can be fully activated via the canonical inflammasome pathway. Nat. Commun..

[36] Rahman T., Nagar A., Duffy E.B., Okuda K., Silverman N., Harton J.A. (2020). NLRP3 sensing of diverse inflammatory stimuli requires distinct structural features. Front. Immunol..

[37] Hochheiser I.V., Pilsl M., Hagelueken G., Moecking J., Marleaux M., Brinkschulte R. (2022). Structure of the NLRP3 decamer bound to the cytokine release inhibitor CRID3. Nature.

[38] Maquat L.E. (2004). Nonsense-mediated mRNA decay: splicing, translation and mRNP dynamics. Nat. Rev. Mol. Cell Biol..

[39] Kikuchi-Yanoshita R., Taketomi Y., Koga K., Sugiki T., Atsumi Y., Saito T. (2003). Induction of PYPAF1 during *in vitro* maturation of mouse mast cells. J. Biochem..

[40] Aganna E., Martinon F., Hawkins P.N., Ross J.B., Swan D.C., Booth D.R. (2002). Association of mutations in the NALP3/CIAS1/PYPAF1 gene with a broad phenotype including recurrent fever, cold sensitivity, sensorineural deafness, and AA amyloidosis. Arthritis Rheum..

[41] Blasi E., Mathieson B.J., Varesio L., Cleveland J.L., Borchert P.A., Rapp U.R. (1985). Selective immortalization of murine macrophages from fresh bone marrow by a raf/myc recombinant murine retrovirus. Nature.

[42] Swamy M., Siegers G.M., Minguet S., Wollscheid B., Schamel W.W. (2006). Blue native polyacrylamide gel electrophoresis (BN-PAGE) for the identification and analysis of multiprotein complexes. Sci. STKE.

