# Supplementary material for: The leucine-rich repeat (LRR) domain of NLRP3 is required for NLRP3 inflammasome activation in macrophages
Source: J Biol Chem. 2022 Nov 17;298(12):102717. doi: 10.1016/j.jbc.2022.102717 (PMC9763864; doi:10.1016/j.jbc.2022.102717)
Supplement: Supplemental Figure S1 [file mmc1.pdf]

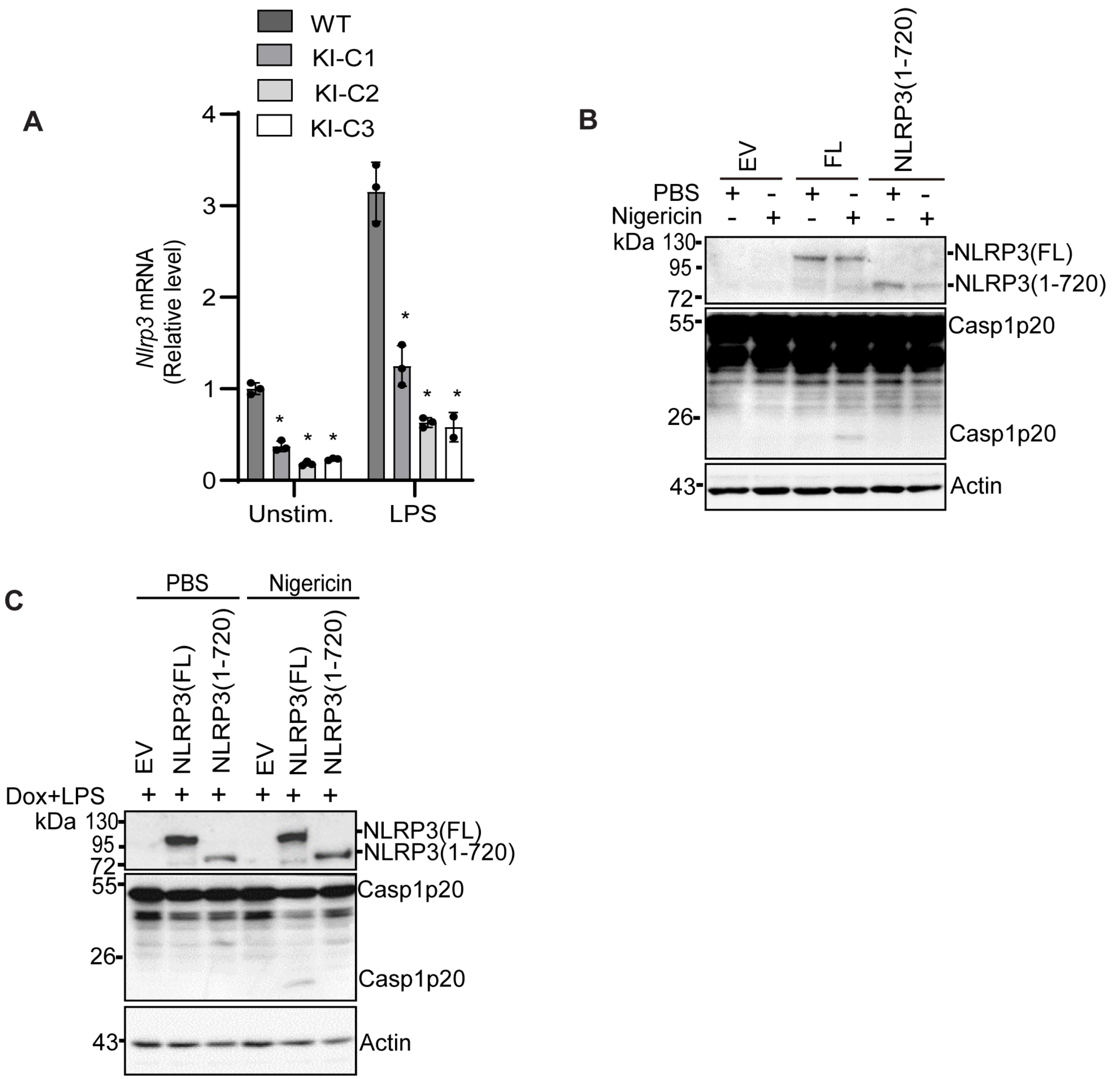

Figure S1 A. Wild-type (WT) macrophages and NLRP3 1-720 knock-in macrophage clones (KI-C1, KI-C2,KI-C3) were stimulated with LPS (200 ng ml^{-1}) for 4 h or left unstimulated. The relative mRNA levels of *Nlrp3* were calculated after quantitative PCR. Data are the mean ± SD of triplicates. * p < 0.05. **B.** Mouse *Nlrp3*$^{-/-}$ macrophages were reconstituted with untagged full-length (FL) or mutant NLRP3 (1-720) and stimulated with PBS (mock) or 5 μM nigericin (1 h) after LPS priming. Mixtures of cell lysates and supernatants were immunoblotted with indicated antibodies.**C.** Mouse *Nlrp3*$^{-/-}$ macrophages were reconstituted with untagged full-length (FL) or mutant NLRP3 (1-720) by a doxycycline-inducible system (pINDUCER21). Macrophages were treated with LPS (100 ng ml^{-1}) and doxycycline (1 μg ml^{-1}) for 12 h before stimulation with PBS (mock) or 5 μM nigericin (1 h). Mixtures of cell lysates and supernatants were immunoblotted with indicated antibodies. Representative blots (n=3). EV, empty vector.
